# Supplementary figures and images for: Transgenic expression of a T cell epitope in Strongyloides ratti reveals that helminth-specific CD4+ T cells constitute both Th2 and Treg populations
Source: PLoS Pathog. 2021 Jul 8;17(7):e1009709. doi: 10.1371/journal.ppat.1009709 (PMC8291758; doi:10.1371/journal.ppat.1009709)

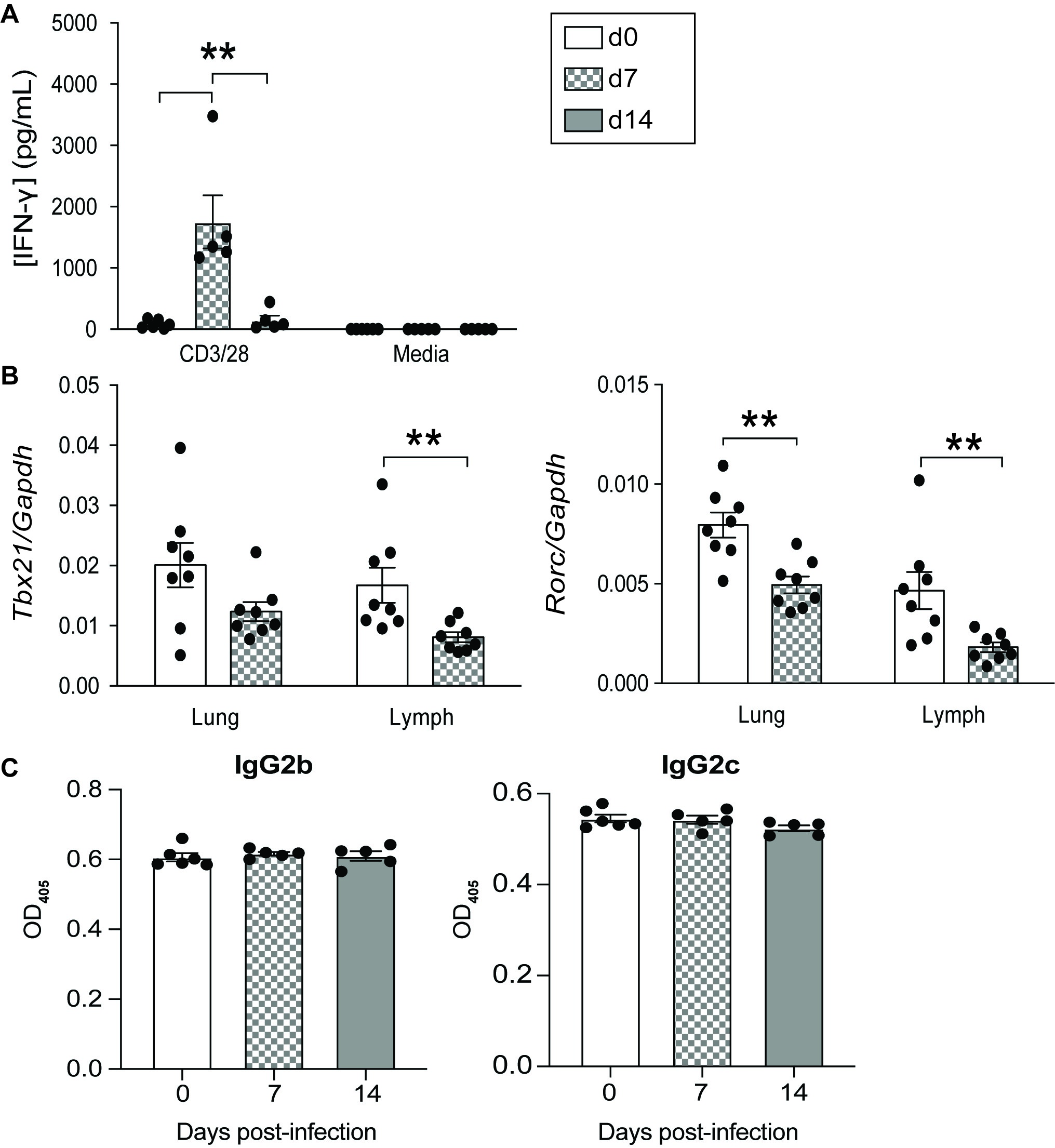

Supplement: S1 Fig — (A) IFN-γ levels in anti-CD3/CD28 (1 ug/mL) stimulated, MACS-sorted CD4+ T cells from secondary lymphoid organs of S. ratti-infected or naïve following 72 hours stimulation (n = 5). (B) Tbx21 (Tbet) and Rorc (RORγt) expression in MACS-sorted CD4+ T cells from lungs and secondary lymphoid organs of naïve and S. ratti-infected mice (n = 8). (C) Total IgG2b and IgG2c antibody absorbance values in sera from naïve and S. ratti-infected mice (n = 5). **p<0.01. (TIF) [file ppat.1009709.s001.tif]

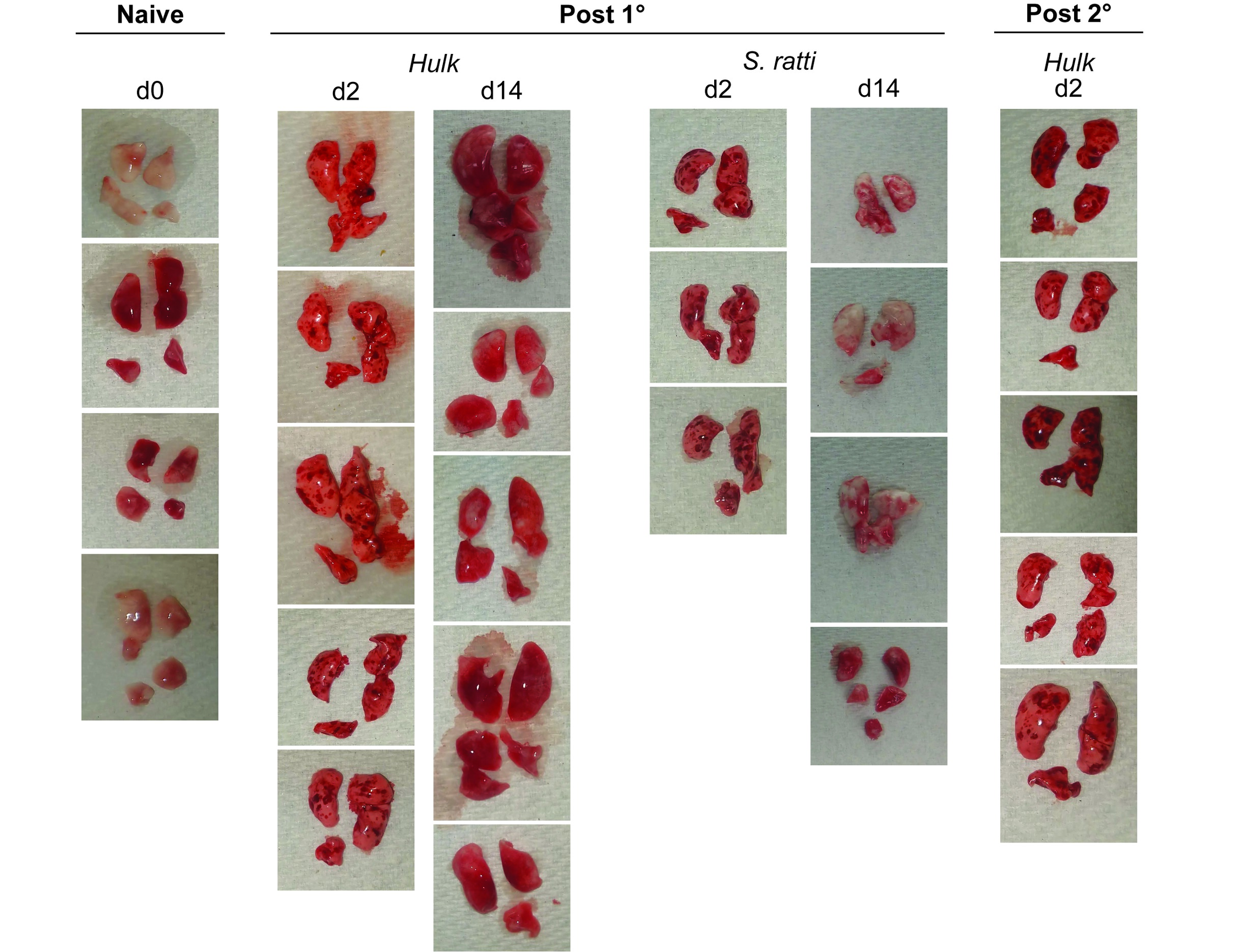

Supplement: S2 Fig — Images of lungs excised from naïve or infected mice at the indicated days post-infection. (TIF) [file ppat.1009709.s002.tif]

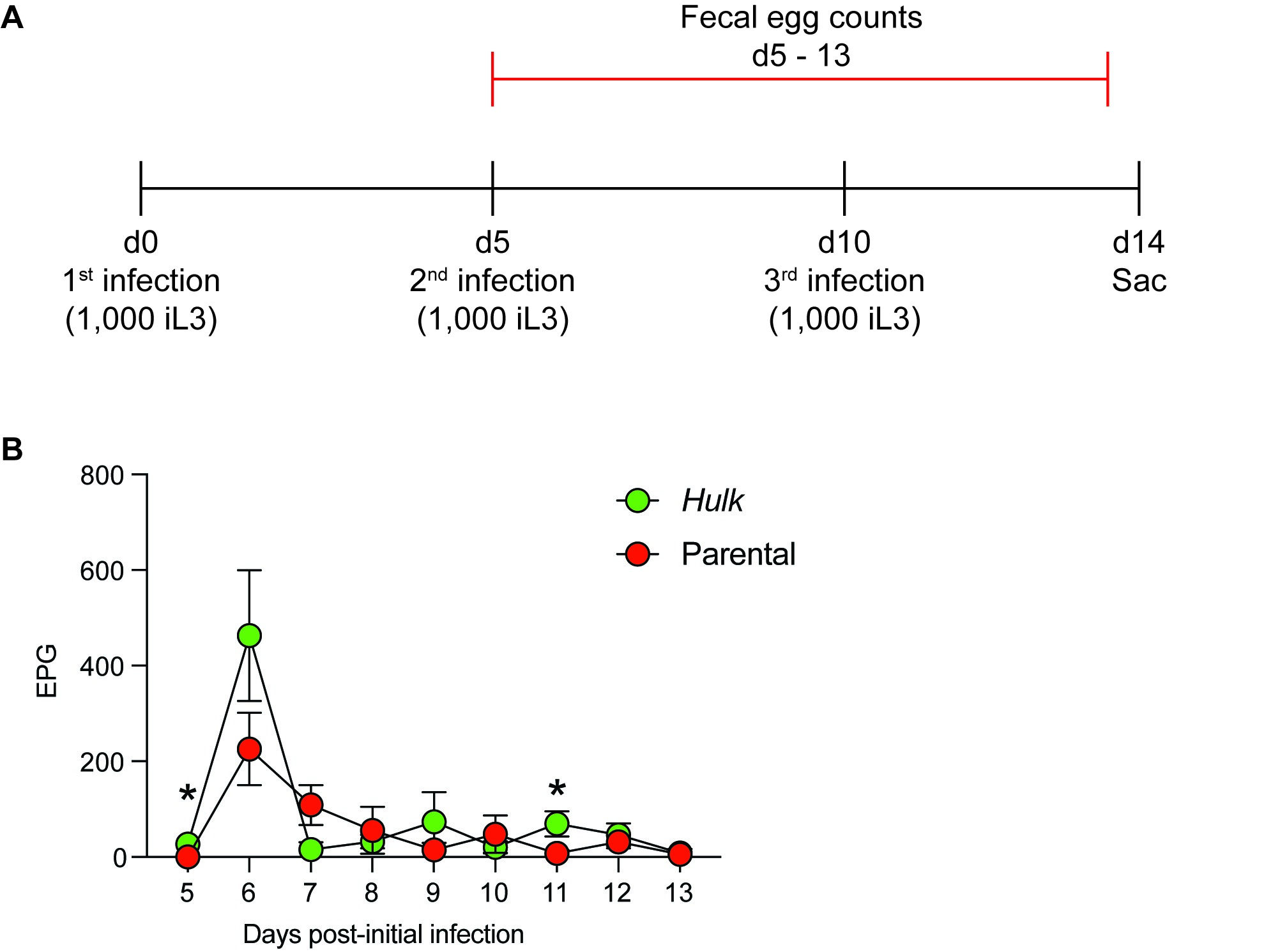

Supplement: S3 Fig — (A) Schematic of experimental layout for 3 infection model. (B) Egg production in feces (denoted as eggs per gram (EPG) over time following 3 infections with Hulk or parental S. ratti. (TIF) [file ppat.1009709.s003.tif]

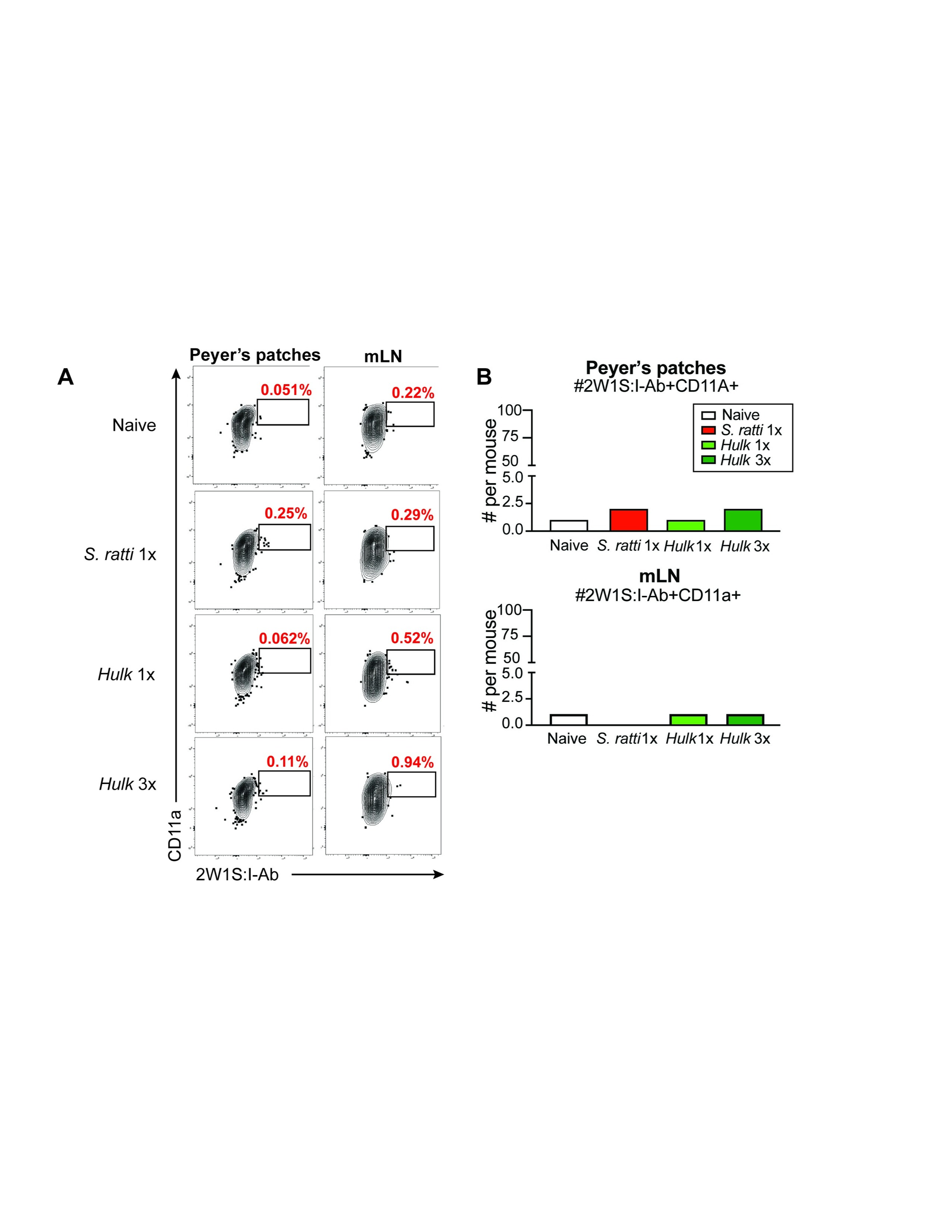

Supplement: S4 Fig — (A) 2W1S:I-Ab+CD11a+ CD4+ T cell frequency in Peyer’s patches and mesenteric lymph nodes (mLN) in naive mice or mice infected once with Hulk or parental S. ratti or three times with Hulk (n = 3–4, pooled). (B) Number of 2W1S:I-Ab+CD11a+CD4+ in Peyer’s patches and mLN in naïve, 1x S. ratti-infected or Hulk-infected, and 3x Hulk-infected mice. (TIF) [file ppat.1009709.s004.tif]

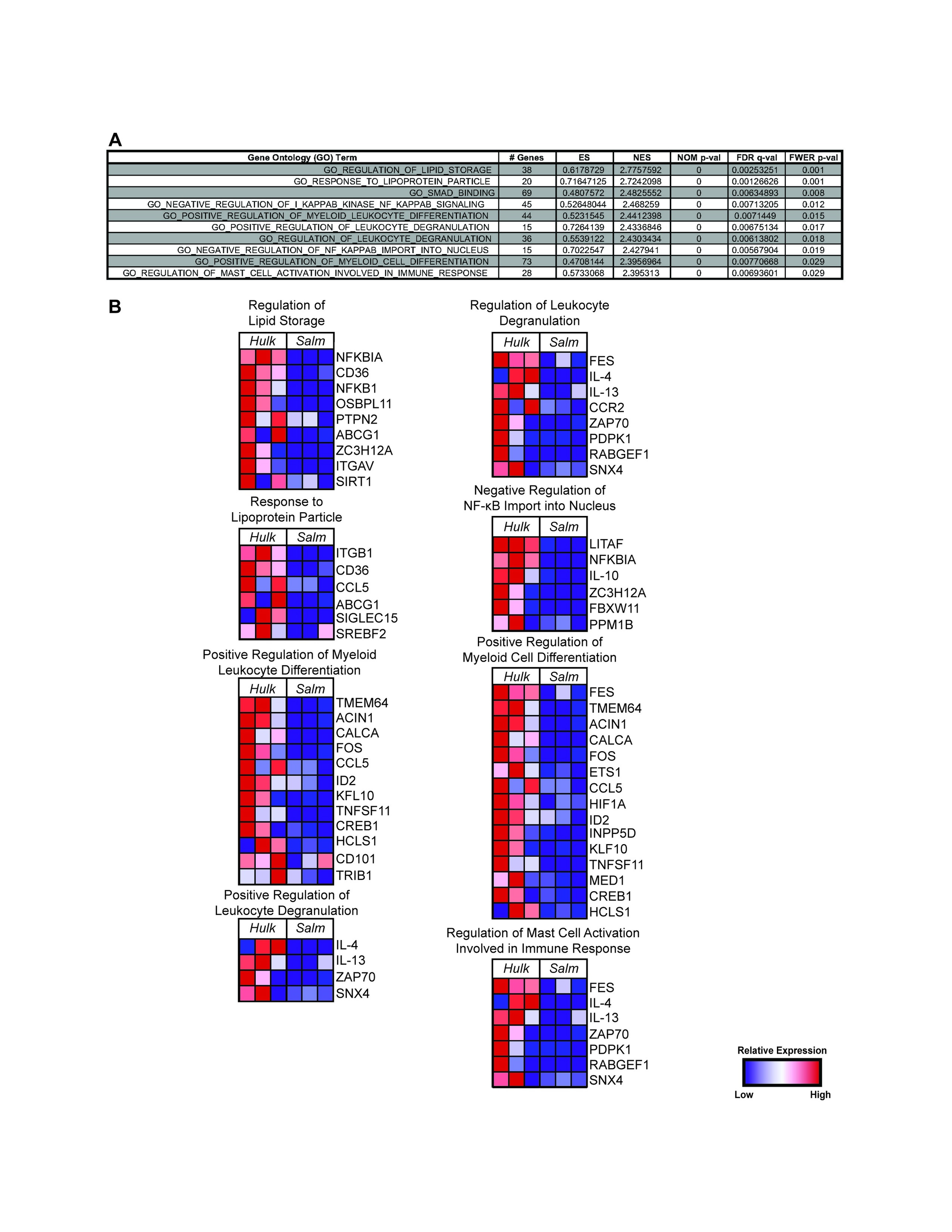

Supplement: S5 Fig — (A) Table depicting the top ten most significantly enriched gene ontology terms within 2W1S+CD4+ T cells from Hulk-infected mice relative to 2W1S+CD4+ T cells from 2W-Salmonella-infected mice. (B) Heat maps showing representative genes upregulated in 8/10 gene ontology pathways upregulated in 2W1S+CD4+ T cells from Hulk-infected mice in addition to those shown in Fig 4. (TIF) [file ppat.1009709.s005.tif]

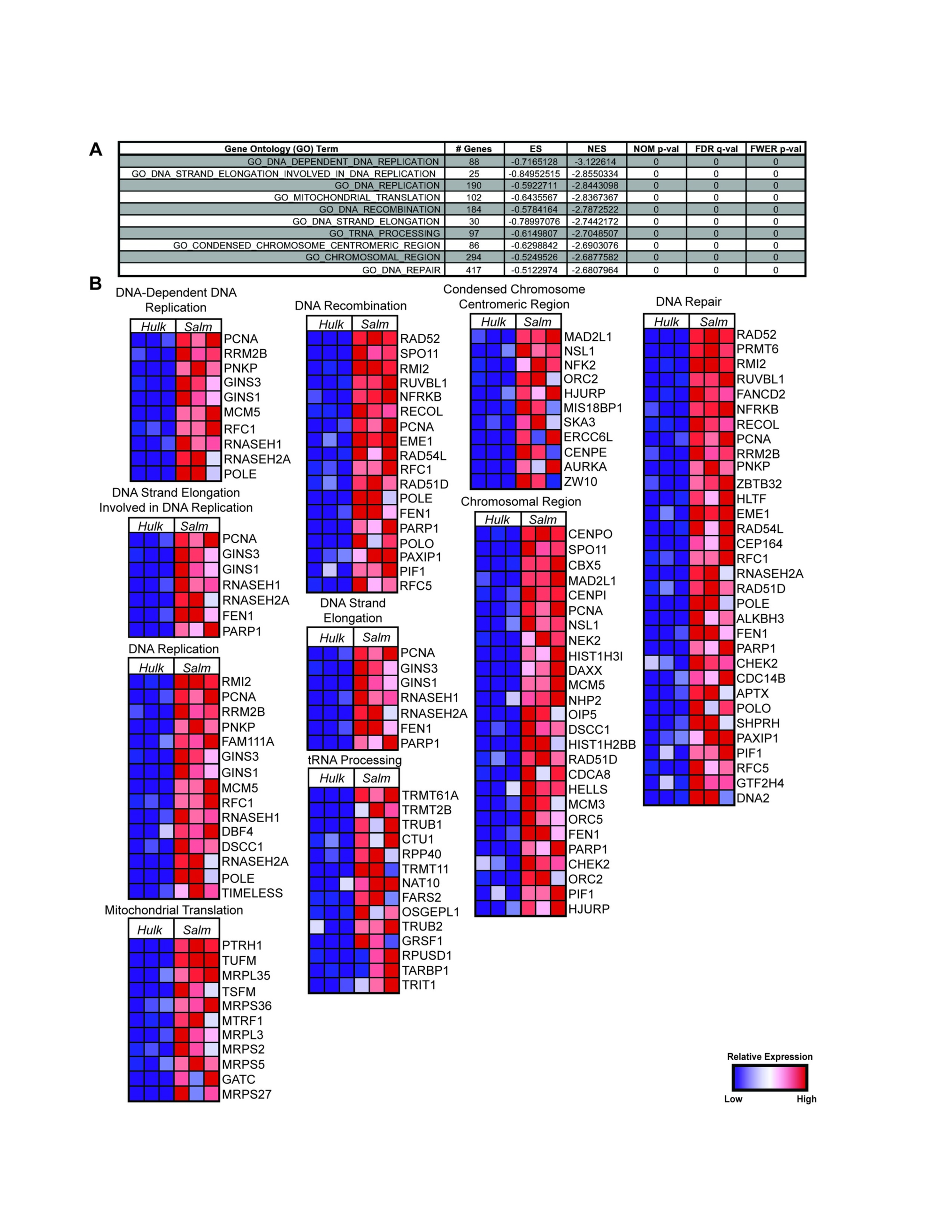

Supplement: S6 Fig — (A) Table depicting the top ten most significantly enriched gene ontology terms within 2W1S+CD4+ T cells from 2W-Salmonella-infected mice relative to 2W1S+CD4+ T cells from Hulk-infected mice. (B) Heat maps showing representative genes upregulated in each gene ontology pathway upregulated in 2W1S+CD4+ T cells from 2W-Salmonella-infected mice relative to Hulk-infected mice in addition to those shown in Fig 4. (TIF) [file ppat.1009709.s006.tif]

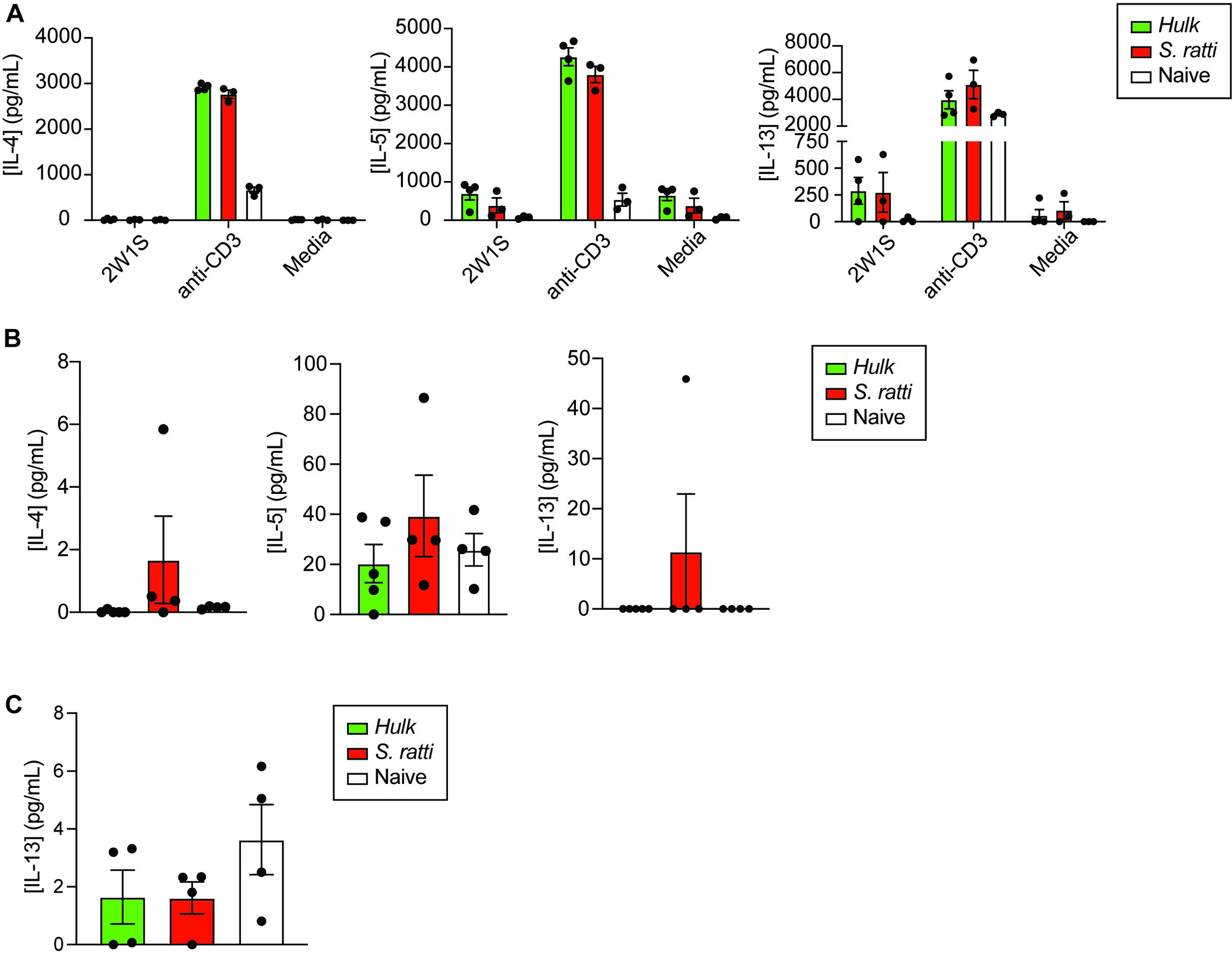

Supplement: S7 Fig — (A) IL-4, IL-5 and IL-13 production by lung cells from naïve mice infected 3 times with live Hulk or parental S. ratti after 72 hours stimulation with 2W1S peptide or anti-CD3. (B) Spontaneous IL-4, IL-5 and IL-13 production by lung cells from naïve mice or mice infected 3 times with irradiated Hulk or parental S. ratti and restimulated with 2W1S peptide intratracheally after 48 hours in vitro culture. (C) IL-13 levels in BALF of naïve mice or mice infected 3 times with irradiated Hulk or parental S. ratti and restimulated with 2W1S peptide intratracheally. (TIF) [file ppat.1009709.s007.tif]

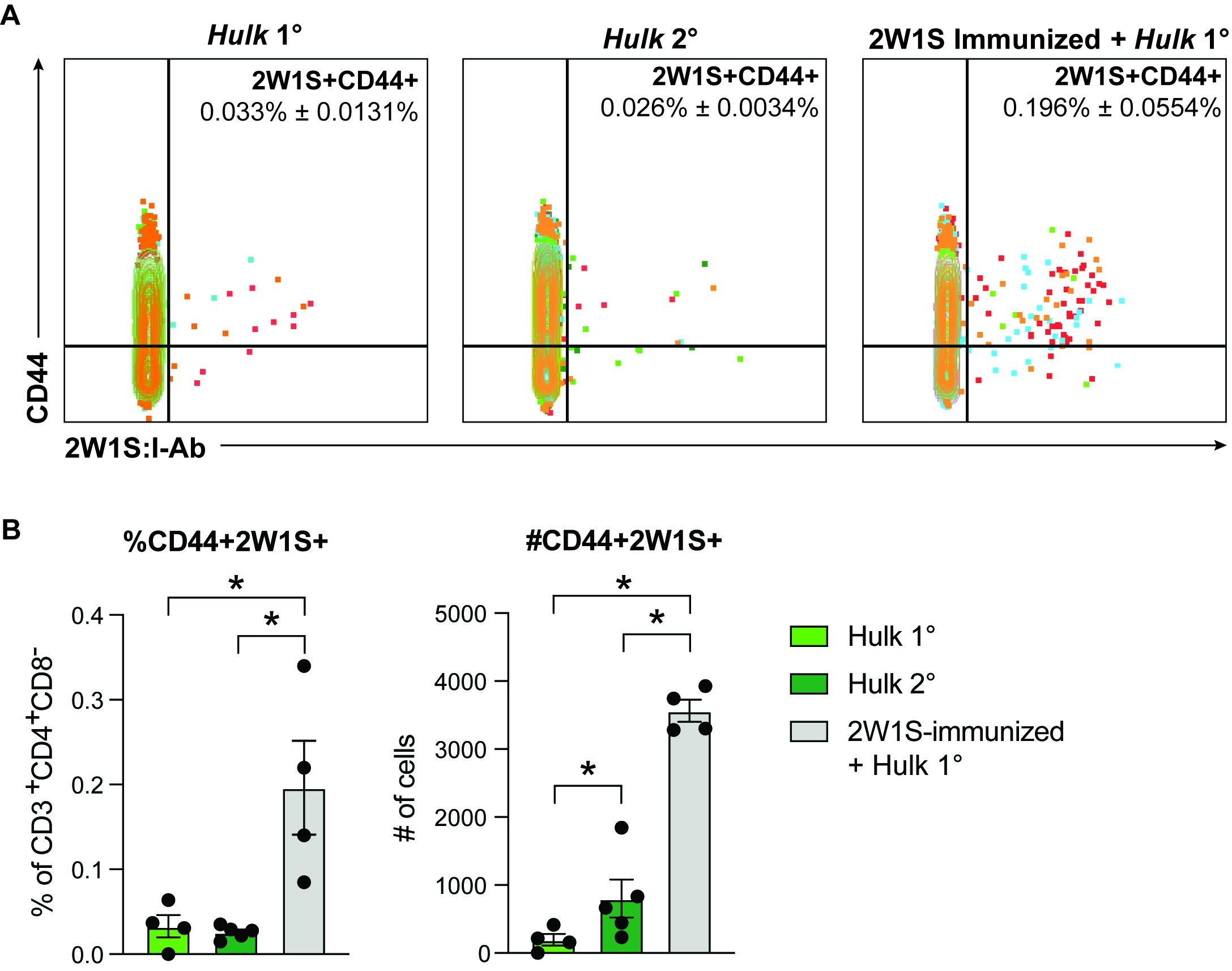

Supplement: S8 Fig — Mice immunized with 2W1S peptide and alum were subsequently infected with Hulk and compared to mice given a primary or secondary Hulk infection. (A) Concatenated flow plots showing the frequency of 2W1S+CD44+ CD4+ T cells in the lungs of each group 6 days post-challenge. Note: Lung cells were not enriched for 2W1S+ cells prior to analysis as in main paper figures. (B) Frequency and number of 2W1S:I-Ab+CD44+ CD4+ T cells in each group 6 days post-challenge. Significance was determined using a Mann-Whitney test; *p < 0.05. (TIF) [file ppat.1009709.s008.tif]

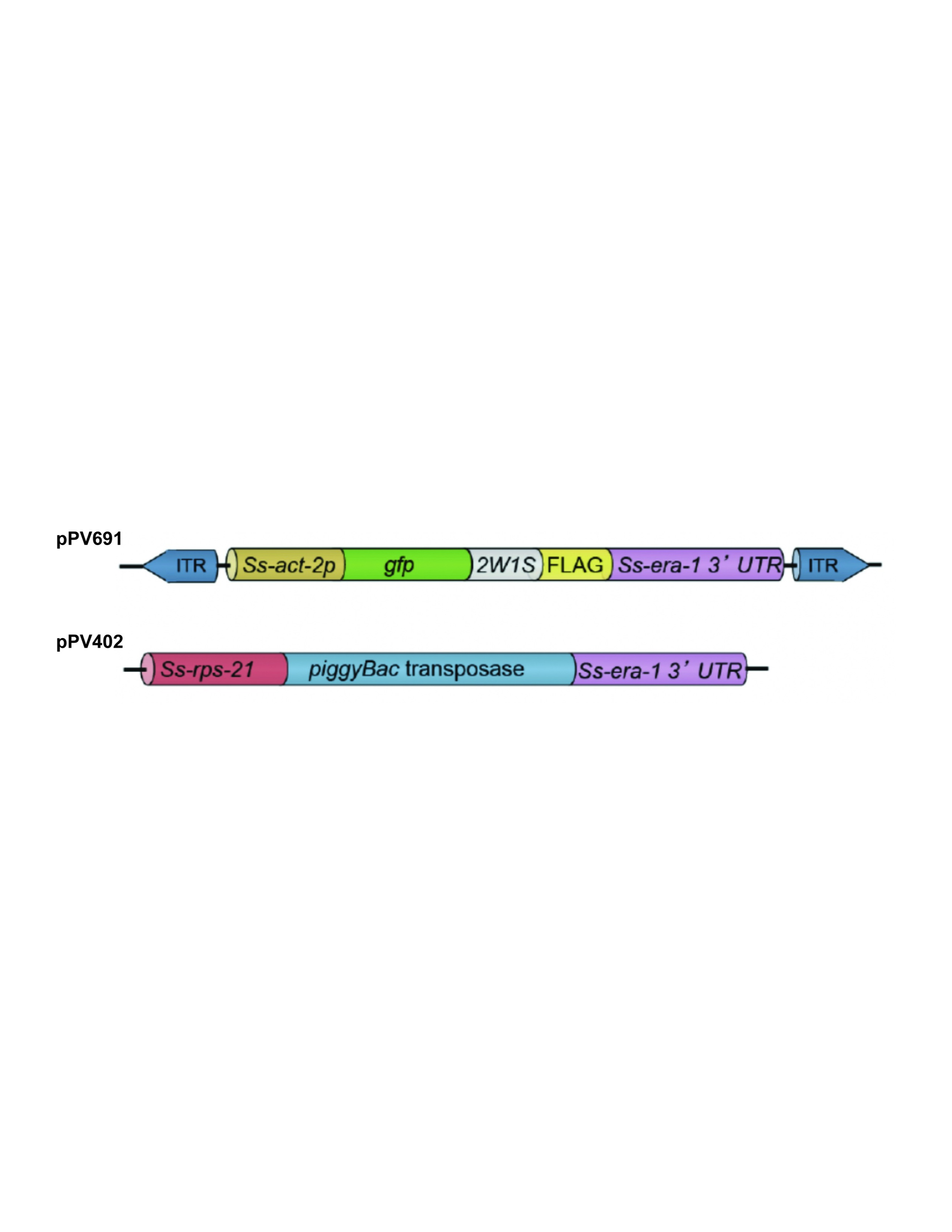

Supplement: S9 Fig — (TIF) [file ppat.1009709.s009.tif]
