# Supplementary material for: Transgenic expression of a T cell epitope in Strongyloides ratti reveals that helminth-specific CD4+ T cells constitute both Th2 and Treg populations
Source: PLoS Pathog. 2021 Jul 8;17(7):e1009709. doi: 10.1371/journal.ppat.1009709 (PMC8291758; doi:10.1371/journal.ppat.1009709)
Supplement: Attachment — Submitted filename: PLOS Pathogens Point-by-Point Reply_v2.docx [file ppat.1009709.s011.docx]

Dear Editor,

Thank you for your consideration of our manuscript. We are very grateful for the feedback that you and the reviewers provided, and we feel it has significantly improved the quality of this work.

Please find attached a revised version of the manuscript, as well as our point-by-point response to reviewer comments below in blue:

****************************************************************************************************

All reviewers are in agreement with the innovation and technical challenge of generating transgenic parasites and investigating antigen-specific T cells, and agree that these findings would be of significant impact to the field of helminth immunologists.

However, Reviewers 1 and 2 raised important concerns that need to be addressed, notably validation of the antigen-specificity of the T cells with regards to function, and validation of transgenic expression in parasites recovered ex vivo.

Based on these concerns:

Major: three added studies are needed to

- investigate T cell antigen specificity by re-stimulation with antigen

We have performed these studies by re-stimulating lung cells *ex vivo* and by administering peptide *in vivo* intratracheally following *Hulk* or parental *S. ratti* infection. Results show significantly increased frequencies of CD11a+Ki67+ and GATA3+Ki67+ CD4+ T cells and elevated amphiregulin production in response to 2W1S peptide in *Hulk*-infected mice relative to parental-infected or naïve controls. These data can be found in Figure 3F-I and Figure 5A-I, and are referenced on page 9 and pages 11-2, respectively. While we did not observe increased production of type 2 cytokines IL-4, IL-5 or IL-13 in response to 2W1S restimulation, this data has been included as a new supplemental figure (S7 Fig) and is referenced on page 12.

- investigate function of the antigen-specific cells e.g. T cell transfers

We acknowledge the utility of CD4+ T cell transfers and agree that this experiment would enhance the impact of our study. Unfortunately, transferring sorted 2W1S-specific CD4+ T cells from *Hulk*-infected mice is not possible with this model due to the prohibitive number of animals that would be required to complete that task.

Instead, we have completed two different experimental approaches to address whether generation of a 2W1S-specific CD4+T cell response would impact the ability of parasitic worms to reproduce or live within the GI tract of its host. First, in an effort to determine whether prior exposure to 2W1S peptide would enhance immunity to *Hulk*, we immunized WT mice with 2W1S peptide with alum as adjuvant and challenged mice with *Hulk* 6 weeks post-immunization. Despite a significantly higher frequency and number of 2W1S-specific CD4+ T cells in the immunized group than in the unimmunized *Hulk*-infected group, there was no difference in worm burden 6 days post-infection compared to the primary infection group (Fig i). Notably, mice given a second-ary *Hulk* infection did have significantly fewer worms in the intestine at this time point, indicating enhanced protect-ion.

**Figure i. Immunization with 2W1S peptide increases 2W1S-specific CD4+ T cell expansion in response to *Hulk* infection, but does not enhance worm clearance**. Mice were immunized with 100 ug 2W1S peptide and 1 mg alum (2W1S-immunized + *Hulk* 1°) or infected with 1,000 iL3 *Hulk* (*Hulk* 2°). After 6 weeks, mice were challenged with 1,000 iL3 *Hulk*. A naive group of mice was also infected with 1,000 iL3 Hulk as a primary infection control (*Hulk* 1°). (A) Concatenated flow plots showing the frequency of 2W1S:I-Ab+CD44+ CD4+ T cells in each group 6 days post-challenge. Note: 2W1S:I-Ab+ cells were not enriched prior to analysis as in main paper figures. (B) Frequency and number of 2W1S:I-Ab+CD44+ CD4+ T cells in each group 6 days post-challenge. (C) Adult worm counts in the small intestine 6 days post-challenge. Significance was determined using a Mann-Whitney test; *p < 0.05.

**A**

**B**

**C**

Secondly, we have completed experiments wherein 1 x 10^6^ polyclonal *Hulk*-specific CD4+ T cells from previously infected WT mice were adoptively transferred intra-venously into highly suscep-tible RAG1-/- mice (n = 4). After 3 days of reconstitution, these RAG1-/- recipients were infected with live *Hulk* parasites to ascertain whether the polyclonal pool of TCR specificities generated in *Hulk* infected WT hosts could confer protection against infection in an otherwise highly susceptible T and B cell-deficient host.

Our data show reductions in egg production between days 6-8 post-infection and elevated lung IL-4 cytokine levels in RAG1-/- mice after transferring 1 x 10^6^ polyclonal CD4+ T cells relative to PBS recipients. However, by day 9 post-infection, there is no significant difference between CD4+ T cell recipients and PBS-treated controls (Fig ii). We interpret these results to indicate that a minimum of 1 x 10^6^ CD4+ T cells are required to observe a modest and transient reduction in worm burdens in susceptible RAG1-/- recipients.

Thus, to do the experiment requested, which was to transfer only 2W1S- specific cells, there is a technical and ethical issue, because we can only acquire an average of 150 2W1S+CD4+ T cells from the lungs of one *Hulk*-infected mouse. Therefore, if the target is 1 x 10^6^ CD4+ T cells, we would need:

1,000,000 2W1S+CD4+ T cells NEEDED / 150 2W1S+CD4+ T cells per mouse = 6,667 donor mice

In order to make this study reproducible, we would need to transfer cells into at least 4 RAG1-/- recipients, which would require:

4 recipients x 6,667 donor mice = 26,668 donor mice

Obtaining, infecting, and proc-essing tissues from this many mice is both financially and technically untenable.

**Figure ii. CD4+ T cell transfer reduces egg production at peak infection and increases IL-4 production in the lungs of *Hulk*-infected RAG1-/- mice.** CD4+ T cells (1 x 10^6^) were sorted from *Hulk*-infected wild-type mice 7 days post-infection and transferred intravenously into naive RAG1-/- mice. A separate group of RAG1-/- mice received the same volume of cell-free PBS. After 3 days, all mice were infected with 1,000 iL3 *Hulk*. (A) Egg production in feces over time. (B) IL-4 production in supernatant of lung cells cultured overnight +/- 1 ug/mL phorbol 12-myristate 13-acetate (PMA) and ionomycin. Significance determined using a Mann-Whitney test; *p < 0.05, **p < 0.01.

Altogether, we take these findings to mean that a CD4+ T cell response directed against 2W1S is not sufficient to enhance protective immunity against *Hulk*. We cannot not rule out the possibility that a CD4+ T cell response against a different, single endogenous helminth antigen could confer partial or sterilizing immunity against infection.

- validate that worms recovered from the infected mice express the epitope

We have isolated and performed Western blots to detect GFP and FLAG in both the lung-dwelling L4 and gut-dwelling parasitic adult stages of *Hulk*. While we can detect GFP protein consistently in all life stages, FLAG peptide levels are diminished in L4 and undetectable in parasitic adults. Taken together with data showing expansion of 2W1S-specific CD4+ T cells in the lung and mediastinal lymph node, but not gut-draining lymphoid tissue, we interpret these findings to mean that the 2W1S antigen is likely not expressed by the parasitic adult stage. This is an important caveat to this model. These data have been added to Figure 2C-F, and are referenced on pages 7-8.

Without these added data, the conclusions of these studies may be over-interpreted, and do not sufficiently increase our understanding of mechanisms of immunity to intestinal nematode infection. These added revisions would greatly increase the significance and impact of this study.

Minor: Reviewers 2 and 3 requested a better understanding and/or discussion of the kinetics of parasite infection in lung and gut.

We have included additional data addressing L4 and adult worm burdens in the lung and small intestine, respectively, during infection (Figure 2H-I, page 8). We have also included two new supplemental figures (S2 and S3 Figs) showing gross pathology in the lung at 2 and 14 days post-infection and egg production kinetics during the 3 infection model. These data are referenced on page 8 and page 8-9, respectively.

**Part I – Summary**

Reviewer #1: This is an interesting study that uses a transgenic nematode parasite to investigate CD4+T cell specific responses in vivo. Transgenesis of parasitic nematodes has been and continues to be extremely challenging technically. It is noteworthy, therefore, that this study has been able to generate such parasites and investigate immune responses to them in vivo. This is a clear strength of the study. The experiments have been carefully carried out and the analysis is sound and the data novel. However, a weakness of the work lies in that the S, ratti model in the mouse, although it has been extensively worked upon over many years, is quite variable and limited in scope. Infectivity - as defined by a productive intestinal infection - for the mouse is generally very low and variable. The, data , as presented has some deficiencies that need to be addressed. This impacts on the conclusions that can be draw from the data presented in the manuscript at the present time.

Reviewer #2: In this manuscript the authors first evaluate the T cell response that is initiated following a Strongyloides infection. During this analysis they show that infection promotes a mixed TH2 and Treg response that is characterized by the production of type 2 cytokines, IL-10 and amphiregulin. Next, the authors show that lymphocyte responses appear to play an import role in limiting parasite levels by infecting Rag K/O mice, which have elevated parasite loads. To better investigate the role of T cells in response to infection, the authors then generated a 2W1S-GFP-FLAG expressing parasite (Hulk). When mice are infected with hulk antigen-specific T cell responses are observed in the lungs, but not so much in the gut. Transcriptional analysis of these antigen-specific T cells illustrated that they have a strong TH2 cell signature mixed with a Treg signature characterized by high expression of amphiregulin. This differed substantially from T cells generated by SW1S expressing salmonella that promoted more proinflammatory T cell responses.

Reviewer #3: The paper by Douglas et al. is focused on identifying the role of CD4+ T cells in helminth infection. They begin by providing background of the importance of CD4+ T cells in controlling and clearing nematode parasites of the GI tract, and that the nature of CD4+ T cells specific for helminth antigens is unexplored. They developed a wonderful new resource to study helminth infection by producing S. ratti that expresses 2W1S, GFP, and FLAG, all on the body wall of the nematode. The production of this resource required herculean effort, which is not lost on this reviewer. They present compelling data that S. ratti infection elicits CD4+ T cells which produce diverse inflammatory and immunomodulatory cytokines. They presented nice data on the development of their transgenic nematodes, confirming that the they have a stable transgenic line. They then used this new tool to study the role of infection context on the phenotype of CD4+ T cells, demonstrating that 2W1S-expressing Salmonella infection yields a different expansion of CD4+T cells than 2W1S-expression S. ratti.

Overall the paper is well written, the experimental design and progression is logical and clearly conveyed. This study represents a significant advancement to the field and will be of interest to a broad readership. I had only a few minor suggestions. Well done!
 
**Part II – Major Issues: Key Experiments Required for Acceptance**

Reviewer #1: The data (Fig1) from infection of mice with WT S.ratti parasites shows that a CD4+ T cell response (and Type 2) occurs in the lungs on day 14 and at day 7 in site draining pooled lymph nodes. What are these nodes? Are they a mixture of lung draining and gut draining? The data from Fig1F suggests that in RAG null mice the mean intestinal worm burden is approximately 30 worms. Although no data is presented for the WT mice, it is reasonable to assume that it would be a similar number. This is 3% of the infection dose of 1000iL3. Where are all the other parasites?

The pooled lymph nodes assessed in Figure 1 were mediastinal, cervical, mesenteric and inguinal lymph nodes. The inguinal lymph nodes drain the flank skin where iL3 are initially injected. After penetrating or being injected into the skin, *S. ratti* larvae migrate through both the nasal cavity and the lungs, which are drained by the cervical and mediastinal lymph nodes, respectively. The mesenteric lymph nodes drain the gastrointestinal tract, where *S. ratti* adults reside before they are cleared. This information has been added to the Methods section under “CD4+ T cell culture” on page 22 of the revised manuscript.

We and others^1^ have consistently observed attrition in larvae and adult worms recovered from the lungs and small intestine, respectively, relative to the number of larvae inoculated. New data added to Figure 2 (panels H, I) speaks to this point, and are referenced on page 8 of the revised text.

In Figure 2H, mice were infected with 3,000 iL3 subcutaneously, but only 200-300 L4 could be recovered from the lungs 2 days post-infection – representing about 10% of the initial inoculum. Similarly, in Figure 2I, only 50-100 adult worms could be recovered from the small intestines of mice that had been infected with 1,000 iL3 7 days prior, representing 5-10% of the initial inoculum. These data are referenced on page 8 in the text.

This attrition can be explained in a few ways:

1) Some larvae arrest in the skin, and do not make it into the bloodstream^1^,

2) In addition to the lung, *S. ratti* larvae can be recovered in the naso-frontal region of the head and in muscle tissues from infected mice^1,2^. Larvae could arrest or be trapped at all of these sites before reaching the gut,

3) Not all larvae that successfully migrate to the gut establish infection, and those that do are progressively cleared. This is evidenced by work showing that adult worm counts peak as early as 5 days post-infection and wane gradually up to 10 days post-infection^1^. Our data also demonstrates that, while roughly 5-10% of the original inoculum can be recovered as adult worms in the intestine 7 days post-infection, few if any worms can be found by 14 days post-infection. Clearance of worms from the gut is dependent upon both innate immune cells, namely mast cells, and adaptive immune responses^3-5^.

In Fig 2D epg levels are shown for both WT parasites and transgenic parasites which are similar. This is taken as evidence that the WT and transgenic parasites are similar in terms of virulence. Worm burden data was not presented which would have been informative. However, it is not clear if the parasites that received transgenic parasites were indeed transgenic. This is important as the authors say that virtually all but not all the transgenic iL3 that are prepared are indeed transgenic. As the infection level of the WT infection is so low, how can the authors be sure that the worms in the gut are transgenic and expressing the Salmonella epitope? Data showing this would seem to be critical. Also, could the authors provide data of transgenic parasite infection kinetics in Rag/null mice? This would give confidence showing that a productive intestinal infection of transgenic parasites can occur in mice and that the responses seen in the gut are not due to non transgenic parasites. This also has impact on the observation suggesting that there are almost no 2W1S- specific T cells in the gut draining lymph node (S2 Fig) yet apparently transgenic parasites in the intestine (Fig 2D):draining lymph node responses are clearly evident after a similar infection level of WT parasites are present (Fig1). So this begs the question of why there are no transgenic parasite specific T cells detected if parasites and antigen are present.

First, we have added a comparison of worm burdens in mice infected with parental or *Hulk S. ratti* in Figure 2I on day 7 post-infection (referenced on page 8 in text). Though there is a slight increase in adult worms in the parental-infected mice, this difference is not statistically significant and has not been consistently observed.

As requested, Figure 1F shows kinetics data of transgenic parasite (*Hulk*) infection in RAG1-/- vs. WT mice. These data are referenced on page 7 of the manuscript.

The point about transgene expression in parasitic life stages of *Hulk* is salient, and has helped us better characterize this model. New data has been added to Figure 2 (panels C-F) showing that, while GFP can be detected in the L4 and adult worm stages, FLAG expression wanes as *Hulk* develops through its host. Although there is not an antibody against 2W1S peptide to confirm loss of the epitope, we think the FLAG Western blot data – in tandem with the lack of 2W1S-specific CD4+ T cell expansion in gut-draining lymphoid tissue – suggests that the 2W1S epitope is not present in the gut. These data are referenced on pages 7-8, as well as in the Discussion on page 14.

The analysis of responses in the lung tissue (and draining lymphoid tissue) would suggest that there is an anti-transgenic parasite CD4+ T cell response. Sometimes the number of cells is extremely small (less than 10 cells per mouse) and it is difficult to be convinced of significance. The authors also state that there is considerable attrition of the larvae between lung to gut. The data suggests that there is a strong lung response and it is more robust after multiple infections. Is the response more robust if the larval innoculum was increased threefold and given in one dose? Is the response due to a large number of possibly dying parasites ? Does an innoculum of dead transgenic parasites generate a strong CD4+ T cell response? The authors discuss the potential role of parasite specific CD4+ T cell lung responses in host protection, but is is unclear if this is to live parasites bearing in mind the inefficiency of the infection. Why is day 14 chosen as the date for analysis? Most of the parasites will have have gone even from the intestine at this point and much earlier from the lungs?

We believe three infections at different timepoints is necessary for the enhanced 2W1S-specific response in the lung. In support of this, we see that even when mice are given 3 inoculations of 1,000 iL3 on days 0, 5 and 10 post-initial infection, only one peak of egg production is observed around day 6-7 post-infection (S3 Fig, pages 8-9). This suggests to us that the inflammatory environment established by the first inoculation may enhance antigen load in the lung by preventing emigration out of the lung to the gut. Further, while 3 doses of 1,000 live iL3 elicited increased amphiregulin production in response to 2W1S peptide in lung cells from *Hulk*-infected mice relative to controls (Figure 5A, page 11), infection with a single dose of 3,000 iL3 did not yield the same response.

Regarding the utility of dead larvae at initiating a response: we do not think that infecting mice subcutaneously with dead larvae will lead to a greater response in the lung as dead larvae cannot migrate out of the skin. However, our *in vivo* restimulation experiments have utilized irradiated iL3, which are live upon infection but arrest in the lung, to enhance the 2W1S-specific response (Fig 3F-I, page 9 and Fig 5B-I, page 12). Compared to peptide restimulation following live infection, we are able to see more robust expansion of proliferating Ki67+ CD11a+ and GATA3+ CD4+ T cells and production of amphiregulin in response to 2W1S peptide after infecting mice with irradiated iL3. These results suggest that increasing the number of larvae that remain in the lung and, by extension, the antigen pool, likely enhances the 2W1S-specific response.

We initially chose to analyze 2W1S-specific CD4+ T cell responses on day 14 because that is a clearance timepoint. Further, this timepoint allowed us to simultaneously analyze B cell/antibody responses, which we have not found to be robust at earlier timepoints. While it is true that larvae are no longer in the lung by day 14, T cell responses are known to lag slightly behind infection kinetics. Further, we have analyzed 2W1S-specific CD4+ T cell responses in the lung as early as day 7, and in general we find comparable numbers of 2W1S+CD4+ T cells at this site on day 7 as at day 14. Further, day 14 falls only 4 days after the final infection of larvae in the 3x infection model – meaning that the last dose of larvae will have only just migrated out of the lungs at this timepoint. Again, S3 Fig shows a schematic of this infection model as well as infection kinetics by egg production (referenced on pages 8-9).

Reviewer #2: This manuscript addresses a topic of great interest. The ability to track antigens specific T cells following a Strongyloides infection offers substantial benefits. It allows one to test vaccine strategies, to evaluate how long the T cells persists, to test the functional qualities of those T cells in otherwise naive mice, and to evaluate how those responses evolve following during trickle infections. Unfortunately the study appears very underdeveloped in its current form. The majority of the data presented is limited to characterizing the T cell responses and provides virtually the same insight as the analysis of the polyclonal T cell assays shown in Figure 1 (a mixed TH2/Treg induction). Given that the strength of this study was to determine the importance of antigen specificity, it was a bit surprising that the T cells were stimulated with anti-CD3 and anti-CD28 rather than antigen.

We have performed both *ex vivo* and *in vivo* restimulations of *Hulk* and parental-infected mice, as well as naïve mice, with 2W1S peptide. These experiments demonstrated that cells from the lungs or bronchioalveolar lavage fluid (BALF) of *Hulk*-infected mice produce increased amphiregulin relative to controls. Further, *in vivo* restimulation with 2W1S increases the frequencies of proliferating (Ki67+) CD11a+ and GATA3+ CD4+ T cells in the lungs and BAL of *Hulk*-infected mice but not parental-infected or naïve controls. These data can be found in Fig 3F-I (referenced on page 9) and Fig 5A-I (referenced on pages 11-12).

The authors nicely show that antigen specific T cells increase dramatically following multiple infections, but never test how protective those T cells are if they are transferred to naive animals that are later challenged. This manuscript can be advances in many ways that offer substantial insight into the many questions that the authors have mentioned in the introduction and discussion. While the generation of the model is a great advance, it needs to be used to ask questions that reveal more insight than what has already been determine by studying bulk T cell responses.

Please see the data provided in Figures i and ii on pages 2-3 of this rebuttal. We agree that T cell transfers would be informative, but do not think they would be technically feasible with this model. Further, given results from peptide immunization experiments, we do not think these cells are sufficient to confer protection against subsequent infection with such a complex pathogen.

However, we think that the new data we include from 2W1S peptide restimulation in Figure 5A-I further emphasizes the role of these 2W1S-specific CD4+ T cells as Th2 cells and producers of amphiregulin. As explored in our discussion, we propose that these cells may play a role in tissue repair in addition to or instead of in protective immunity.

Reviewer #3: There were no major issues that I identified. The study was well designed and the conclusions are supported by the data presented.

**Part III – Minor Issues: Editorial and Data Presentation Modifications**

Reviewer #1: None

Reviewer #2: (No Response)

Reviewer #3: In the section on the effects on Hulk on 2W1S-specific CD4+ T cell expansion, the authors comment (195-201) that the lung parenchyma holds the predominance of the 2W1S-specific CD4+ T cell response. I was glad to see that in the discussion they mentioned that it is likely that lung-resident APCs have greater access to nematode body wall components. This discussion could be expanded to include mention of the number of infecting IJs, how many make it to the lung, and how many end up in the gut. But that’s not a necessary addition.

We have added new data in Figure 2H-I which speak to this point. These data are referenced on page 8 of the revised manuscript.

Line 126: I’m a little uncertain as to the use of “head cavity” in describing where the nematodes go after infection. I’ve just never heard that term used to describe the trachea or esophagus, but more in reference to the cranial cavity.

The phrase “head cavity” refers to the naso-frontal region of the head, rather than the trachea/esophagus. This phrase has been replaced with “naso-frontal region of the head” in the text (page 5)

Line 280: change to “is mediated by lung-resident…”

This change has been applied and can be found on page 15.

Line 326: This is the first mention of DSS colitis and the meaning of “DSS” is not explained

This change has been applied and can be found on page 16-17.

**References:**

1. Dawkins HJ, Grove DI. Kinetics of primary and secondary infections with *Strongyloides ratti* in mice. *Int J Parasitol*. 1981 Feb;11(1):89–96.

2. Tindall NR, Wilson PA. Criteria for a proof of migration routes of immature parasites inside hosts exemplified by studies of *Strongyloides ratti* in rat. *Parasitology*. 1988 Jun; 96(pt 3):551-63.

3. Reitz M, Brunn M-L, Rodewald H-R, Feyerabend TB, Roers A, *et al*. Mucosal mast cells are indispensable for the timely termination of *Strongyloides ratti* infection. *Mucosal Immunol*. 2016 July;10:481-92.

4. Reitz M, Hartmann W, Rüdiger N, Orinska Z, Brunn M-L, Breloer M. Interleukin-9 promotes early mast cell-mediated expulsion of *Strongyloides ratti* but is dispensable for generation of protective memory. *Sci Rep*. Jun 2018;8:8636.

5. Meiners J, Reitz M, Rüdiger N, Turner J-E, Heepmann L, *et al*. IL-33 facilitates rapid expulsion of the parasitic nematode *Strongyloides ratti* from the intestine via ILC2- and IL-9-driven mast cell activation. *PLoS Pathog*. Dec 2020;16(12):e1009121.
