# Supplementary material for: Transgenic expression of a T cell epitope in Strongyloides ratti reveals that helminth-specific CD4+ T cells constitute both Th2 and Treg populations
Source: PLoS Pathog. 2021 Jul 8;17(7):e1009709. doi: 10.1371/journal.ppat.1009709 (PMC8291758; doi:10.1371/journal.ppat.1009709)
Supplement: Attachment — Submitted filename: PLOS Pathogens Point-by-Point Reply 2_v2.docx [file ppat.1009709.s012.docx]

Dear Editor,

Thank you once again for your thoughtful feedback on our manuscript. Please find attached a newly revised version of our manuscript, as well as a point-by-point reply to reviewer comments below in blue:

On resubmission, both reviewers agree that the authors have made efforts to address their original comments but there is concern that the data provided in the response suggest significant technical difficulties in using these transgenic parasites to investigate the function of antigen-specific T cells due to their limited number and the lack of data demonstrating that they provide protection. Based on these comments:
1. Text changes are recommended to (i) remove the language "revolutionize", and (ii) add a discussion of the caveats of this model including low numbers antigen-specific T cells making functional transfer experiments prohibitive, and the caveats of the S.ratti infections in mice.

(i) We have changed “*This constitutes a technological advancement that could potentially revolutionize the field of helminth biology*” to “*This constitutes a technological advancement that offers new possibilities for the study of antigen-specific immune responses during helminth infection*” on page 19.

(ii) Caveats of this model have been outlined in the Discussion section on page 15.

2. Per recommendation of Reviewer 2, please add Figure (i) to the manuscript as a supplemental or main figure with additional data assessing of amphiregulin expression or tissue repair in the lungs of these groups of mice.

We have demonstrated that amphiregulin-expressing CD3+ cells are significantly increased in the lungs of 2W1S peptide-immunized mice 3 days post-*Hulk* infection relative to mice given primary or secondary infections alone. We have also shown that total amphiregulin production by lung cells 3 days post-infection is elevated in mice that received 2W1S peptide immunization compared to non-immunized primary and secondary controls. These findings, along with worm burden data from immunized and non-immunized primary-infected and secondary-infected mice, have been added to the manuscript in Figure 6 and are referenced in the Results on page 13-14. Additionally, we have added data showing the frequencies and numbers of 2W1S-specific CD4+ T cells in the lungs of these three groups of mice in Supplemental Figure 8, which is also referenced on pages 13-14 of the revised manuscript.

At this time, we do not have sufficient data to adequately address whether 2W1S-specific CD4+ T cells ameliorate lung damage or promote tissue repair. However, we have included preliminary data which speaks to this point in Figure i on page 2 of this rebuttal, and are planning to follow-up on this question in the future.

Reviewer Comments (if any, and for reference):

Reviewer's Responses to Questions

**Part I - Summary**
Please use this section to discuss strengths/weaknesses of study, novelty/significance, general execution and scholarship.

Reviewer #1: The study presents data from an initial study using a transgenic nematode parasite. This is a technological step forward and shows that this approach is doable. The authors provide some interesting data to verify the validity of the approach. The authors have clearly made attempts to address my earlier concerns and conducted extra experiments which have clarified some of the issues that I had raised and made sensible rebuttals to other points.

Thank you, we appreciate your feedback.

Reviewer #2: The authors have done some nice work in the new manuscript and showed that antigen specific activation of the T cells results in areg expression. They also attempted to vaccinate the mice with peptide and looked at worm burdens in the gut. The authors show increased expansion of the T cells but never looked at areg or tissue repair. This needs to be done and they may also want to look in the lung given the previous data shown. This paper is presenting a technical achievement that the authors say "could potentially revolutionize the field of helminth biology in regards to antigen-specific immune responses." The current version of the paper still fails to show how this new model will advance our knowledge of these T cell responses beyond their known functions.

We agree that the language in this sentence of the manuscript was too strong, and have altered this language as described above. This edit can be found on page 19 of the revised manuscript.

However, we argue that the lack of resistance conferred by 2W1S immunization against *Hulk* infection is still a novel and important finding. To our knowledge, this is the first time that expansion of a T cell population specific for an antigen expressed in a pathogen has not conferred any level of resistance against subsequent infection. Moreover, it has never before been possible to study whether T cells specific for a single gastrointestinal nematode peptide epitope promote worm expulsion. We maintain that even the negative results we present in regards to the protective capacity of 2W1S-specific CD4+ T cells in *Hulk* infection are useful to the field.

**Figure i. 2W1S peptide immunization results in a slight reduction in BAL red blood cells (RBC) 3 days post-*Hulk* infection.** Mice were immunized with 100 ug 2W1S peptide and 1 mg alum (2W1S-immunized + *Hulk* 1°) or infected with 1,000 iL3 *Hulk* (*Hulk* 2°). After 3 weeks, mice were challenged with 1,000 iL3 *Hulk*. A naive group of mice was also infected with 1,000 iL3 Hulk as a primary infection control (*Hulk* 1°). Three days post-challenge, RBCs in the BAL were quantified by subtracting cell numbers post-RBC lysis from cell numbers pre-RBC lysis. Significance was tested using a Mann-Whitney test, data pooled from 2 independent experiments.

That said, we have demonstrated an increase in both total amphiregulin levels as well as amphiregulin-expressing T cells in the lungs of immunized, *Hulk*-infected mice (Figure 6, referenced on pages 13-14 of the revised manuscript). Importantly, we have also made efforts to address the question of tissue damage in our immunization system. Following a single immunization with 2W1S peptide, we observe a very slightly reduction in the number of red blood cells in the bronchoalveolar lavage (BAL) of immunized mice 3 days post-*Hulk* challenge, relative to non-immunized primary or secondary *Hulk*-infected mice (Figure i). This finding may be associated with a mild reduction in lung damage in immunized mice, and we are planning to follow up on this finding in subsequent work. As suggested, multiple immunization boosters may further ameliorate tissue damage or promote tissue repair – but this requires additional optimization. Given the data we have at this time, we do not feel comfortable stating that 2W1S-specific CD4+ T cells reduce tissue damage or contribute to tissue repair at later timepoints post-*Hulk* challenge.

I'm not in agreement that a single experiment of 1M T cells into a Rag animal allows you to determine that 26,000 mice would be needed to run an entirely different experiment. The idea would be that you have enriched on a highly potent population of T cells that could rapidly expand in response to seeing the antigen in the new host. This is done routinely in other models and is the reason a system like this is so helpful. Even-though the logic of coming to the 26,000 number is confusing it brings to light the concern that this model is not as useful as hoped. This statement was troubling and suggests that the model is not very useful for future experiments that could lead to very exciting results.

We concede the technical limitations of this model and agree that functional analyses are crucial for pushing this field forward. However, we still argue that this work represents a technical innovation and serves as a proof of concept for future studies of antigen-specific CD4+ T cells in helminth infections. Nevertheless, a discussion of the technical caveats of this model has been added to the revised manuscript on page 15.

That authors at least need to show they can detect changes in areg when they vaccinate. Maybe that will require a couple of boosters. The data shown in Figure i should then be added to the study. With data like this added it would show that the model is going to be useful in the future.

We have demonstrated that 2W1S peptide immunization a) increases total amphiregulin production by lung cells by ELISA and b) increases the number of CD3+ and Areg+CD3+ cells in the lung parenchyma following *Hulk* infection relative to mice given primary or secondary infection alone. This data is shown in Figure 6C-F, and is referenced on pages 13-14 of the revised manuscript.

**Part II – Major Issues: Key Experiments Required for Acceptance**
Please use this section to detail the key new experiments or modifications of existing experiments that should be absolutely required to validate study conclusions.

Generally, there should be no more than 3 such required experiments or major modifications for a "Major Revision" recommendation. If more than 3 experiments are necessary to validate the study conclusions, then you are encouraged to recommend "Reject".

Reviewer #1: None.

Reviewer #2: (No Response)

**Part III – Minor Issues: Editorial and Data Presentation Modifications**
Please use this section for editorial suggestions as well as relatively minor modifications of existing data that would enhance clarity.

Reviewer #1: In the discussion - lines 336-349 the authors discuss the differences in expression of the transgene and that it is lost from the adult parasitic stages. Given the nature of the discussion it would be useful to briefly discuss this in relation to the expression of free living adult female worms, which clearly do express the transgene (Fig2B). In the last paragraph of the discussion I am not sure we are at the stage of "revolutionizing" the field of helminth biology quite yet.

We appreciate the suggestion regarding transgene expression in free-living vs. parasitic adults, and have addressed this point on page 16.

We agree that the language in this sentence of the manuscript was too strong, and have altered this language as described above. This edit can be found on page 19 of the revised manuscript.

Reviewer #2: (No Response)

We greatly appreciate the time and effort that you and the reviewers have given to improving our work, and we look forward to your feedback on this revision.

Sincerely,

De’Broski R. Herbert
